# Young Adults’ Intentions and Rationales for COVID-19 Vaccination Participation: Evidence from a Student Survey in Ho Chi Minh City, Vietnam

**DOI:** 10.3390/vaccines9070794

**Published:** 2021-07-16

**Authors:** Quy Van Khuc, Trang Nguyen, Thuy Nguyen, Linh Pham, Dang-Trung Le, Hong-Hai Ho, Tien-Binh Truong, Quoc-Khai Tran

**Affiliations:** 1 Vietkaplab, Hanoi 100000, Vietnam; quy.khucvan@vietkap.com; 2 School of Banking-Finance, National Economic University, Hanoi 100000, Vietnam; 11207216@st.neu.edu.vn; 3 Department of Economics, University of Central Oklahoma, Edmond, OK 73034, USA; lpham17@uco.edu; 4 Real-Time Analytics, Ho Chi Minh 700000, Vietnam; trungle@rta.vn; 5 Faculty of Business and Economics, Phenikaa University, Hanoi 12116, Vietnam; hai.hohong@phenikaa-uni.edu.vn (H.-H.H.); binh.truongtien@phenikaa-uni.edu.vn (T.-B.T.); 6 U Minh Ha National Park, Ca Mau 970000, Vietnam; vqguminhha@gmail.com

**Keywords:** COVID-19, vaccination participation, students, Ho Chi Minh city

## Abstract

The COVID-19 pandemic, a source of fear and anxiety worldwide, has caused many adverse impacts. Collaborative efforts to end COVID-19 have included extensive research on vaccines. Many vaccination campaigns have been launched in many countries, including Vietnam, to create community immunization. However, citizens’ willingness to participate is a prerequisite for effective vaccination programs and other related policies. Among all demographic groups, participation rates among young adults are of interest because they are an important workforce and are a source of high infection risk in the community. In March 2021, a pool of approximately 6000 participants in Ho Chi Minh City were randomly polled using an email-based online survey. The exploratory results of 398 valid observations show that students’ perceptions of the dangers of COVID-19 and the importance of vaccination were both relatively high (4.62/5 and 4.74/5, respectively). Furthermore, 83.41 percent of students polled (*n* = 332) chose vaccination, while 16.59 percent chose hesitation (*n* = 64) and not to be vaccinated (*n* = 2). More importantly, our estimated results of the Bayesian regression model (BRM) show that the perceived importance of the vaccine, concerns about the vaccine’s side effects, and a lack of access to information are the top three reasons for their reluctance and/or refusal to get vaccinated. These findings are a valuable resource for politicians, researchers, and those interested in COVID-19 vaccinations to devise and execute campaigns to effectively combat this terrifying pandemic.

## 1. Introduction

Vaccination is deemed the most effective method for containing the COVID-19 pandemic. According to Centers for Disease Control (CDC), all approved vaccines for COVID-19 are proven to demonstrate 65–95% efficacy in clinical trials against COVID-19 in adults aged over 18, and vaccination has shown its effectiveness against COVID-19 infection and symptomatic diseases in the real world in a range from 64% to 99% [1]. Moreover, real-world data also show consistency with findings from clinical trials and indicates reduced risks of COVID-19 hospitalization as for adults aged 65+ thanks to vaccination [2]. Upon vaccine inoculation, people in the community can return to their everyday life, as the reduction in confirmed cases eases the burden of preventive measures and social distancing policies. Thus, vaccines help protect people and those around them [3], and from an economic and medical viewpoint, financial resources allocated to vaccination programs are much safer and much less costly than disease treatment and social distancing. The COVID-19 pandemic has impeded the world’s sustainable economic growth. The BBC states that the IMF’s estimated reduction in the global economic activity is 4.4% [4], and according to Global Economic Prospects, the global GDP is forecast to experience a contraction of 5.2% in 2020 [5].

Without a doubt, vaccines enable high protection against COVID-19, a number of vaccination programs have sprung up worldwide to guarantee the well-being of individuals and communities. To maximize the effectiveness and efficiency of vaccination efforts, it is important for policymakers and the government to understand the public’s perception of COVID-19 vaccines. Research demonstrates a positive relationship between awareness and actions [6], where more positive perception increases the level of willingness to be involved in vaccination programs. As a result, it cannot be denied that research into the perception of people about vaccination can provide a guideline for policymakers to take proper measures to turn citizens’ awareness into actions, thereby increasing the participation rate of vaccination programs if necessary.

Having effectively controlled the pandemic, Vietnam has a relatively low infection rate in comparison to many other countries in the region and around the world [7]. As of 18 May 2021, only 37 deaths and 4579 infected cases were recorded [8]. Vietnam actively promotes vaccination, but the number of people vaccinated remains relatively small, accounting for less than 1% of the population. Limited awareness and reluctance to participate in vaccination among target groups including young people are a challenge for vaccination programs. Deputy Minister of Health Do Xuan Tuyen claimed that within the remaining 3 to 6 months of 2021, the estimated figure for doses of COVID-19 vaccines per day was about 300,000–500,000 [9]. There is also an increasing number of working people getting vaccinated against COVID-19, especially those in industrial areas [10,11,12,13]. This is not only an important labor force but also a potentially dangerous source of infection for the community because of active social lives and the high level of mobility, such as study, work, and tourism activities, compared to other groups. In fact, during the first outbreaks in Hanoi, female patient number 17 was also in this age group [7,14]. In this sense, this study aims to learn about young adults’ attitudes and perceptions toward certain vaccines and their willingness to participate in the COVID-19 vaccination in Ho Chi Minh city, the largest and most populous city in the country. The study also investigates why they are not willing to take the COVID-19 vaccines.

The remaining sections are organized as follows. Part 2 presents an overview of research on COVID-19 vaccination and the young population. Part 3 provides the research methodology, including study site selection, sample size justification, and data analysis methods. Parts 4 and 5 describe the empirical results and discussions, respectively. The last section draws conclusions along with possible policy implications.

## 2. Literature Review

In this section, we present a summarized review on residents’ decisions on vaccination in various countries. More importantly, we highlight several factors that influence vaccination intention, which facilitates not only identifying candidate variables for the empirical model construction in Section 3.3, but also identifying our contributions to the literature.

### 2.1. Vaccination Rate Heterogeneity across Countries

This paper adds to the literature regarding the willingness of the public to receive a COVID-19 vaccine. Kadoya et al., (2021) [15] studied the COVID-19 vaccine acceptance rate in Japan and found that only 47% of respondents were willing to take the vaccine. Biasio et al., (2021) [16] found that the majority (90%) of Italian adults were willing to take the COVID-19 vaccines. Dodd et al., (2021) [17] studied the willingness to vaccinate against COVID-19 in Australia and found that 85.8% of the surveyed respondents were willing to get vaccinated when they become available, and the willingness to get the vaccine is positively correlated with health literacy and the education level. Guidry et al., (2021) [18] analyzed the willingness to get COVID-19 vaccines using a sample of 788 adults in the US and found that the perceived benefits of the vaccines, age and race/ethnicity were among the main determinants of COVID-19 vaccination. This study also shows that concerns about rushed vaccine development lower the willingness to get vaccinated. McPhedran and Toombs (2021) [19] analyzed the determinants of COVID-19 vaccination in the UK and showed the importance of vaccine efficacy in determining the selection of COVID-19 vaccines. This finding is consistent with the findings of Harapan et al., (2020) [20], who studied the COVID-19 vaccination in Indonesia. Cahapay (2021) [21] studied the willingness of Philippine teachers to vaccinate against COVID-19 and found that the majority of teachers were uncertain about whether they should vaccinate against COVID-19.

### 2.2. Individual Characteristics Influence Vaccination Decisions

Many studies identify individual characteristics such as gender as a determinant of vaccination decisions; however, the findings so far have been mixed. While some researchers primarily expected no significant relationship between gender in COVID-19 vaccine acceptance, many studies revealed that males seem more hesitant to get vaccinated than females [22]. According to Jeffrey V. Lazaru and her contemporaries, women in France, Germany, Russia, and Sweden were more likely to receive the COVID-19 vaccination than men [22]. In contrast, the studies [23,24,25,26,27,28,29] revealed that females had higher odds of opting out of COVID-19 vaccines than males. They showed that female respondents often choose “no” or “not sure” over “yes” [24]. In a piece of research in Israel, as regards the willingness to take part in a vaccine trial, the differences were largely between males and females, where females were more likely to reject COVID-19 vaccines [25]. It could be said that the COVID-19 vaccination acceptance between genders remains unclear, but gravitates towards males. Thus, further study is needed to clarify the gender differences in the willingness to get COVID-19 vaccinated.

Another factor that influences the vaccination hesitance and/or acceptance is perception, for example, whether a person or a group has an adequate knowledge of COVID-19 vaccines or has misperceptions due to misinformation can influence vaccine acceptance or hesitancy [30]. The previous study revealed that knowledge about the COVID-19 pandemic and its impacts have a moderate, positive relationship with vaccination. In terms of the severity of the COVID-19 pandemic, the studies [23,31,32,33,34,35] found that individuals who were fearful of getting infected with COVID-19 viruses were more inclined to receive COVID-19 vaccines than those who were not. Consistently, Md Abul Kalam and his contemporaries (2021) also concluded that the severity of a COVID-19 infection was significantly correlated with vaccination uptake [36]. Acceptors of vaccination were feeling 1.3 times as serious about the pandemic as non-acceptors [36], while Mohammed Al-Mohaithef found the same results with the gap of 2.13 times between acceptors and non-acceptors [32].

Regarding individual trust in government policies, in the study of Patricia Soares et al., (2021) [37], individuals who found the information provided by health authorities inconsistent and contradictory had higher odds of refusing to get vaccinated than those who found the information clear and understandable. Respondents in the study [32] who said they trusted the health system were 3.05 times as likely to accept the vaccine as those who said they did not. Besides, those who adhered to government regulations such as wearing masks, social distancing, or lockdown were more willing to participate in COVID-19 vaccination than others who did not [38].

### 2.3. The Effects of the Pandemic and Vaccination Decisions

A study measuring the effects of COVID-19 in people’s daily habit [37] indicated that individuals who felt agitated, sad, or anxious due to the physical distancing measures on some days were more willing to get vaccinated than those who did not. Besides, the costly expenditure that COVID-19 brought us was also taken into people’s consideration. Md Abul Kalam found that acceptors of the vaccines were 1.3 times more likely to believe vaccination may help reduce this exorbitant cost than the non-acceptors [36].

### 2.4. Vaccine Cost and Quality and Vaccination Decisions

Vaccine quality is deemed an essential determinant [23,39] in the decisions of opting for COVID-19 vaccination. Some research found out that people who were concerned about the efficacy of vaccines likely have higher odds of refusal or hesitation about taking them [37,39], while others who believed vaccines were safe were more likely to get vaccinated [36]. Besides, in terms of side effects, subjects who were willing to accept the COVID-19 vaccine expressed less concern about the side effects in comparison to those who opted out of vaccination against COVID-19 [35,36,40]. Using Chile as a case study, García and Cerda (2020) [40] find that public acceptance toward the COVID-19 vaccine depends on the efficiency of the government at handling the pandemic. These previous statements support a positive relationship between vaccine trust and decisions to get vaccinated. Thus, an idea to improve vaccination proportions is to provide thorough information about the vaccines to build trust with the citizens, also recommended by Patricia Soares [37].

Jagdish Khubchandani indicated that when the additional condition such as “if it was free or covered by health insurance” was added to the question, the vaccine acceptance level increased [41]. This implies that the cost of vaccines is also a determinant leading to the willingness to participate in vaccination. Logically, the study [30] revealed that some people might show willingness to get vaccinated, but they could not afford the vaccine price or the costs associated with getting to the immunization point. Furthermore, the negative relationship between out-of-pocket costs of vaccination and vaccine acceptance indicated that respondents preferred vaccines with lower out-of-pocket costs, suggested by [42], so to expand vaccination coverage, immunization programs should be designed to remove barriers in terms of vaccine price and other costs [23].

Table 1 below provides a summary of the recent literature on COVID-19 vaccination. A common theme in the above literature is that there is substantial heterogeneity in the cross-country vaccination acceptance rate and its determinants Sallam (2021) [43]. This implies that the results of a demographic group in a country cannot be generalized to those in another country, and findings of different demographic groups are shown to vary even within a country. To this end, this paper contributes to the literature by being the first study to explore the willingness to vaccinate against COVID-19 among Vietnamese students.

## 3. Materials and Methods

### 3.1. Study Area

Among provinces and cities in Vietnam, HCMC students were chosen as the research target to collect data from because the city’s characteristics and current states of affairs fit our research purposes and requirements. HCMC is the most densely populated city in Vietnam, with a population of about 8.99 million people [48], It is also a large metropolis that builds intimate relationships with many other cities and countries, greatly influencing the Vietnamese economy. Thus, the city faced a high risk of a pandemic outbreak [49]. When the third wave of COVID-19 occurred from 27 January 2021 till 21 March 2021 in Vietnam [50], HCMC became a center of the epidemic on 13 February with a high speed of transmission, and it took authorities around a fortnight to get the situation under control [51]. Among all HCMC residents, this study focuses on students, because of that group’s high exposure to public places, leading to a faster spread of the disease. 

### 3.2. Data Collection

Because COVID-19 has led to strict laws on social distancing and restrictions on traveling [7,48,52], online survey methods were applied. During the survey process, we conducted online meetings with the survey team to get updated with work progress and promptly make changes to the questionnaire if problems arose. Online surveys can reach a large number of people within a click, and smartphone users in Vietnam constitute over 45% of the population [53]. This trend is predicted to persist nationwide based on the government policy of universal use of smartphones, which shortens survey time and saves financial resources [54]. To reduce bias, we strove to design a questionnaire that was as logical and concise as possible with a fair number of questions and various means of online survey delivery. 

To optimize survey outcomes and accelerate the survey process, we worked collaboratively with Real-Time Analytics, a company that has a reputation for online survey delivery (https://rta.vn, accessed on 15 July 2021). This company specializes in online research, and our online survey form is sent to their survey participant pool via emails to reach randomly around 6000 target participants. Our team designed the study in two steps. After the questionnaire was formed, we selected a focus group [55] to consolidate interviewers’ understanding about data collection procedures and clear up any confusion about the wording and arrangement of questions necessary to get ready for the final data collection step [55,56]. The final questionnaire consisted of 31 questions designed to collect information in five sections: perceptions and impacts of COVID-19, vaccine awareness, the priority of vaccine origins, willingness to be vaccinated, and respondents’ personal information.

### 3.3. Data Description

We received 665 responses from target participants. After excluding the missing data and questions with double information, 398 valid observations were retained for data processing and analysis. We used SPSS 22 to obtain descriptive statistics including the mean, standard deviation, standard error, minimum and maximum values, range, and a confidence interval of 95% to capture the features of the students’ decisions and attitudes about vaccination, as seen in Table 2. In Table 3, we compare means using two independent sample tests, including the Mann–Whitney U test for the dependent variable, which is ordinal but not normally distributed [57]. It is noted that students are categorized by gender—male and female—which allows us to explore the differences in perceptions of each gender in the COVID-19 vaccination.

### 3.4. Bayesian Linear Regression Model

**Figure 1:**
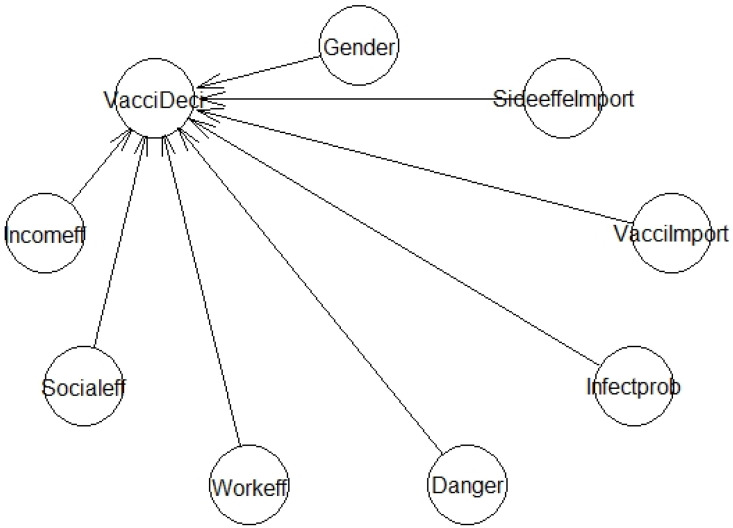
The model of vaccination decision.

We used a Bayesian regression model (BRM) to identify the factors affecting the respondents’ intention to vaccinate (Figure 1). This method has gained popularity among scholars because it does not require large sample sizes or strict assumptions, as the frequentist approach does [58]. It should be noted that Bayesian statistics approaches are increasingly being used by scholars in the social sciences and humanities [58,59]. Following the procedures of [60], we built the BRM using 8 independent variables from 4 major factor groups: characteristics of the respondents, perception about effects, perception about risk, and perceived importance of vaccines. To evaluate and/or validate the model, either *Rhat* or the sample size effect (*n_eff*) metric is used. To be more specific, the model is adequate when *Rhat* equals 1 or *n eff* equals or exceeds a threshold of 1000 [60]. Besides, the MCMC chains for the Bayesian model of Vaccination Decision is also used to evaluate and/or validate the model. Dense densities and/or the consistency of the MCMC chains, for example, indicate a good model. In the following, we will present the model Formula (1), a list of the specific variables used (Table 2), a sampled R code (Box 1), and the visualization of the vaccination decision model (Figure 1). It is noted that the arrow represents the direction from the independent variable to the dependent variable, and the length of the arrow has no meaning.

Model formula:

(1) $$\begin{array}{ll} \mathrm{VacciDeci} & \;\sim \;\mathrm{Incomeff}\;+\;\mathrm{Socialeff}\;+\;\mathrm{Workeff}\;+\;\mathrm{Danger}\;+\;\mathrm{Infectprob}\\ & \;+\;\mathrm{VacciImport}\;+\;\mathrm{SideeffeImport}\\ & \;+\;\mathrm{Gender} \end{array}$$

An example of sampled code (Box 1) that was used to command the Bayesian package in order to implement the hierarchical Vaccination Decision model is shown in the following section.

Box 1 **The design of the model.**

model1a < -bayesvl()

model1a < -bvl_addNode(model1a,”VacciDeci”,”binom”)

model1a < -bvl_addNode(model1a,”Incomeff”,”norm”)

model1a < -bvl_addNode(model1a,”Socialeff”,”norm”)

model1a < -bvl_addNode(model1a,”Workeff”,”norm”)

model1a < -bvl_addNode(model1a,”Danger”,”norm”)

model1a < -bvl_addNode(model1a,”Infectprob”,”norm”)

model1a < -bvl_addNode(model1a,”VacciImport”,”norm”)

model1a < -bvl_addNode(model1a,”SideeffeImport”,”norm”)

model1a < -bvl_addNode(model1a,”Gender”,”binom”)

## 4. Results

### 4.1. Perceptions and Stated Reasons

Table 3 illustrates several aspects affecting respondents’ families and their perceptions of vaccines’ roles during the COVID-19 pandemic. Overall, COVID-19 has taken a toll on human lives in many ways, and students were well aware of vaccine-related issues. Its general impacts on the community’s health and economic life scored 3.42/5, and the likelihood of getting infected with COVID-19 in Vietnam was rated 3.18/5. The research results demonstrate that there was only a slight discrepancy in how this dreadful pandemic affected students’ income, traveling, shopping habits, workplace, and the frequency of meeting with friends (3.34/5). The rate of 4.74/5 suggests that most students surveyed perceived vaccines as a deciding factor for disease containment, and respondents set a premium on effectiveness, 3.96/5. COVID-19′s danger is referred to as threats to human life and the economy, received a high rating of 4.74/5; Vietnam’s success story of responding to COVID-19 contributed to participants’ strong feelings of assurance (4.57/5). It came as a surprise to see that the price of COVID-19 vaccines was relatively highly valued (over 3.8/5), as opposed to their side effects (3.4/5).

**Figure 2:**
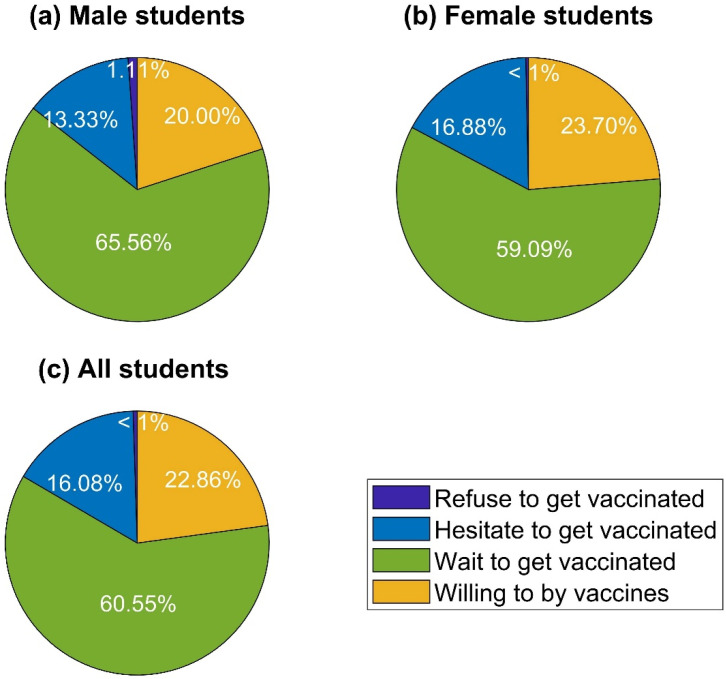
Respondents’ intent to participate in COVID-19 vaccination.

**Figure 3:**
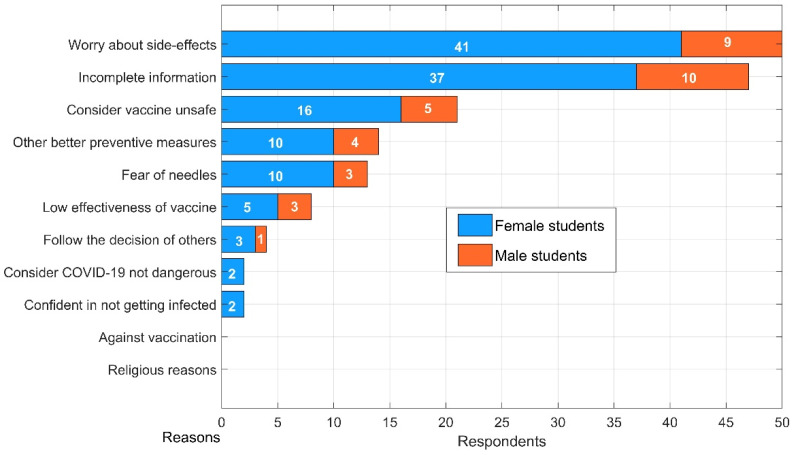
Reasons for hesitation regarding getting vaccinated.

**Figure 4:**
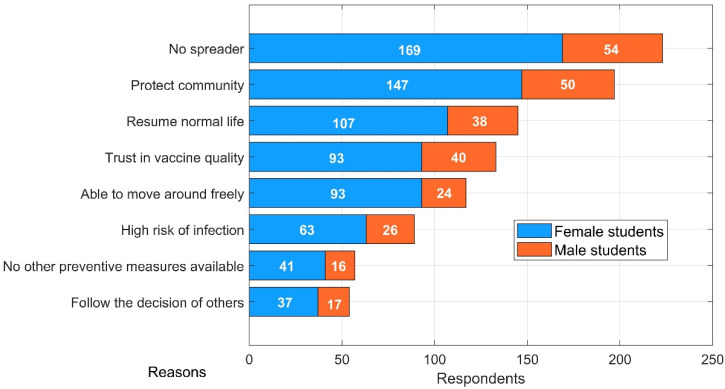
Reasons for getting vaccinated.

Figure 2 illustrates the proportion of students’ choices regarding getting vaccinated against COVID-19. In general, consistent with their perceptions of the importance of vaccines during the COVID-19 outbreak, almost all respondents would opt for vaccines, especially when the vaccines are offered free of charge. In contrast, a negligible percentage of the surveyed people refused to receive COVID-19 vaccines on the grounds of concerns about the side effects and safety. To be specific, free doses of vaccines were recorded as the most favorable option, constituting a hefty proportion of 60.55%. Statistics indicate that roughly one-fourth of the respondents showed their willingness to pay for COVID-19 vaccines, followed by those who hesitated to get vaccinated (16.08%). The bar charts in Figure 3 and Figure 4 provide more insights into students’ decisions about vaccination participation.

Figure 3 presents data on rationales behind students’ indecision over engagement in vaccination programs in Ho Chi Minh City. It can be clearly seen that side effects and incomplete information related to vaccines were considered as significant concerns inducing respondents to defer their decisions. In total, 50 out of 64 Saigonese students’ uncertainty primarily stemmed from their worry about side effects, an attitude that has been well documented on a global scale since the advent of COVID-19 vaccines [15,29,61]. In addition, participation demands a high level of transparency, since 47 respondents hesitated due to incomplete information, which is twice as many as those concerned about the vaccines’ safety. According to our survey findings, the figures for those who feared needles and hoped for better precautions were practically the same. A small number of students did not feel the need for vaccination because no confirmed cases had been reported in their nearby residential areas.

Figure 4 compares different reasons for students’ choices of vaccination participation in HCMC. Overall, contributions to the common good, rather than personal motives, were the main driver of vaccination willingness. Our results indicate that the figures for students who opted to take the vaccine to not be a spreader and protect the community as a whole were 223 and 197, respectively. Interestingly, although vaccine quality is expected to be of paramount importance, it only ranked fourth, with 133 votes. Those who considered vaccination to be an enabler of bringing daily lives back to normal accounted for approximately half of the total survey participants. Nearly one-fifth of students surveyed saw a justification for vaccination participation on the grounds of herd immunity and lightened burdens of preventive regulations. 

Table 4 compares and contrasts the perceptions and vaccination intentions of male and female students. Regarding the impacts of COVID-19 on the students’ lives, although the total impact level recorded no difference between two groups, with a *p*-value of 0.903, there was a considerable disparity in their perception about the infection probability, with a *p*-value of 0.021. Additionally, male and female students are also significantly different in their perception about the peaceful and/or stable level/assurance level (how assured respondents feel) as a resident in Vietnam, at *p*-value = 0.002. It can be inferred from the table that vaccine importance, origin, side effects, and effectiveness were equally valued by both sexes. Awareness of the price and convenience were different in these two genders, with *p*-values of 0.052 and 0.008, respectively. In other words, male is more sensitive to price and convenience than female. To compare the decisions on vaccines of both genders, we assume that those who are willing to pay for vaccines and wait for free vaccines are Group 1, named as getting vaccines, while those who hesitate and reject vaccines belong to Group 0, named as no vaccination. Notably, a comparison of median values of vaccine decisions in both genders using the Mann–Whitney U test showed that there are no differences in the correlation within each gender (*p*-value = 0.536) (see the last row of Table 3) [62].

### 4.2. Determinants of Vaccination Participation Decision

**Figure 5:**
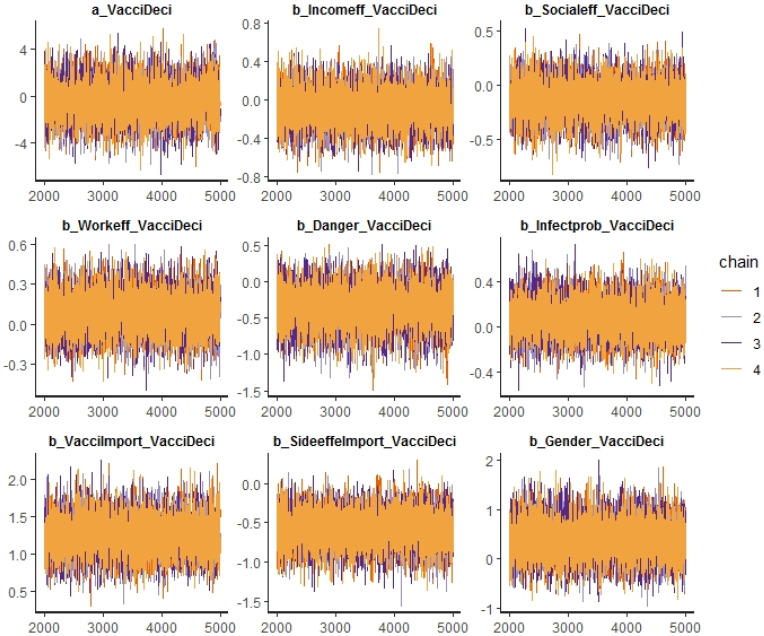
The MCMC chains for the Bayesian model of vaccination decision.

**Figure 6:**
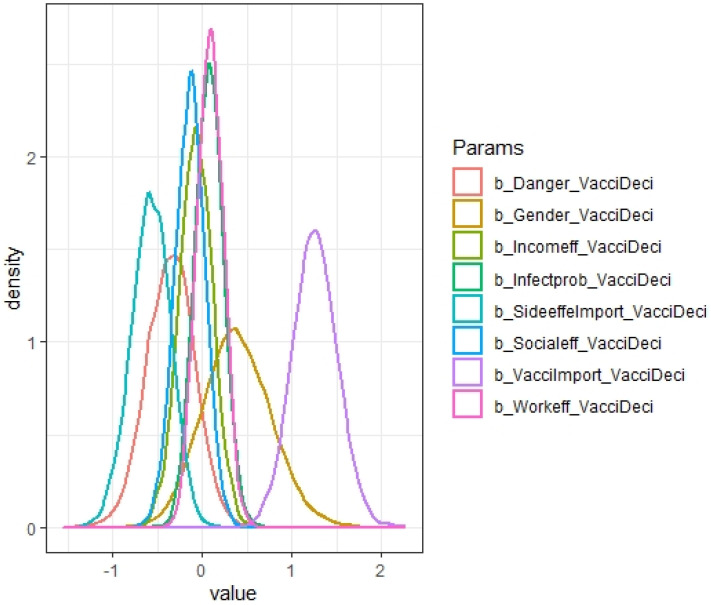
Posterior coefficients of the vaccination decision model.

**Figure 7:**
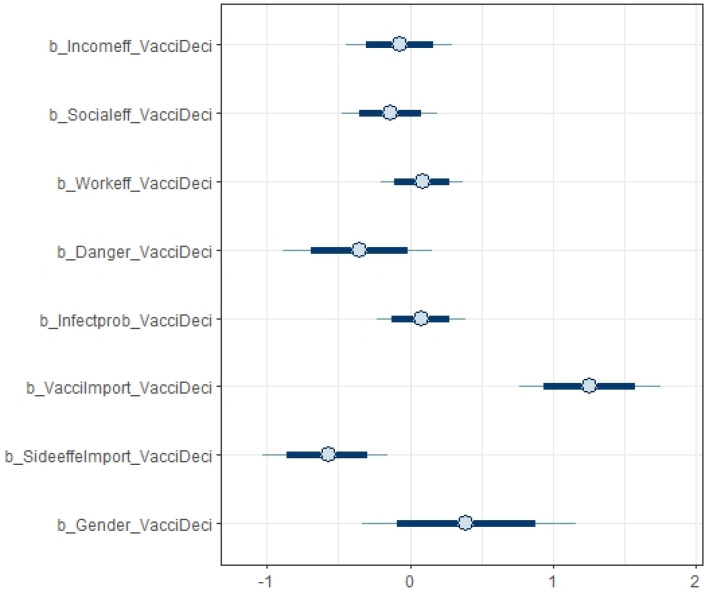
Distribution of the coefficients of factors influencing decisions on vaccination.

Table 5 provides the estimated results of how different factors influenced respondents’ decisions on vaccination using the BRM described in Section 3.4. Specifically, the table provides the summary statistics of the coefficients estimated from the BRM. For all variables, *Rhat* is 1, and *n_eff* is over 7000 (much higher than the threshold of 1000, indicating a good model desired for estimation). Figure 5 shows a high density of plots of variance and ascertains the convergence of our model. The Markov chain Monte Carlo (MCMC) method was used to calculate large hierarchical models in Bayesian statistics. In general, there was consistency among all chains, suggesting the autocorrection phenomenon. In addition, Figure 6 and Figure 7 show the distribution of the coefficients from the BRM. 

In the following, we summarize how various variables influence vaccination decisions, based on the findings of the BRM reported in Table 5 and Figure 6 and Figure 7. Only vaccine side effects and importance of vaccines were shown to be statistically significant, and interestingly, gender does not have a statistically significant impact on vaccination decisions. To be specific, there was a strong positive association between respondents’ perceived importance of vaccines and their willingness to get vaccinated (mean = 1.26). On the other hand, the coefficient of side effects lied within the negative zone, which means that vaccine side effects discouraged people from receiving COVID-19 vaccines (mean = −0.58). As illustrated, the distribution of work-related effects (mean = 0.09) and likelihood of infection (0.07) are narrow with a high density, denoting their firm association with intentions to get vaccines. Although danger and income effects are reported to induce respondents to opt out of vaccination as expected (mean = −0.35 and mean = −0.07, respectively), Figure 5 suggests that these two factors were not statistically significant. 

## 5. Discussions

Detected at the end of 2019, COVID-19 has constantly wreaked havoc on many aspects of human lives. It has served as the culprit causing a significant loss of human life and severe implications for global economic sustainable development, as a huge spike in new infections and deaths is reported on a regular basis. Recently, a new variant of greater danger named B.1.167, originating from India, was found responsible for a new phase of COVID-19 outbreaks in Vietnam. Although some advances in both pharmaceutical and clinical management interventions have been achieved, the world pins its hopes on vaccines to achieve community immunization and eradicate COVID-19.

COVID-19 vaccines are generally believed to be of great benefits, and many people have strong faith in vaccine effectiveness when it comes to immunization and disease control. A survey conducted by CDC suggests that mRNA vaccines have been proven to be 94% effective if patients are fully vaccinated [63]. The National Institute of Hygiene and Epidemiology (NIHE) states that around 30% of vaccine recipients experience mild reactions after inoculation, and it is unlikely that a vaccine can guarantee a 100% safe rate [64]. On 7th May 2021, a 35-year-old female nurse in An Giang died of anaphylaxis due to allergic reactions to non-steroidal anti-inflammatory drugs, which is an extremely rare incident regarding COVID-19 vaccination [65,66]. Mistrust in vaccines is believed to occur accordingly, predictably causing a decrease in residents’ willingness to participate in vaccination programs and a rise in vaccine hesitancy. In response to this, the Vietnamese government can use our dataset as a source of reference to come up with policies promptly to incentivize citizens to opt for vaccines against COVID-19.

The empirical results of our study indicate that HCMC young adults highly value vaccines in terms of eradicating the COVID-19 pandemic. For example, the students’ willingness toward COVID-19 vaccination participation made up a hefty proportion of 83.41%, nearly 30% higher than our expectations. These results on students’ perception about COVID-19 vaccination provide a blueprint for COVID-19 vaccine policies. Our research results are in consistency with some previous research conducted in China [67] and the US [61], where the overwhelming proportion of people agree to participate in COVID-19 vaccination. Our results of a high willingness to be vaccinated (83.41%) in HCMC is relatively higher than other related research, namely, 64.01% in China [67] and 69% in the US [61]. Herd immunity or community immunity requires at least 80% of the whole population to be vaccinated [68,69]. As a result, our research findings denote the potential success of vaccination programs in Vietnam in achieving herd immunity.

Many variables are taken into consideration in our exploration and analysis, but only three variables strongly influence decisions about vaccination: lack of information on vaccines, concern about possible side effects, and perception about vaccines’ importance. Besides, the consistency of the exploratory results and estimated results from BRM indicate that the concerns about vaccine side effects are the most important factors of the vaccination decision. This is highly in line with numerous other studies over the past few years [15,29,70,71,72]. Our research indicates that, in the future, public awareness and access to accurate information about vaccines will require greater communication efforts while much more attention should be paid to the side effect factor of vaccines.

Our study makes many contributions to COVID-19 repulsion by facilitating effective formulation and implementation of vaccination policies. For instance, based on our statistics, policymakers can feel motivated by residents’ satisfaction with governmental countermeasures to better their performance and decide on proper future courses of action. Residents’ willingness is a key factor of successful vaccination policies, and our data provide guidance to devise strategies to promote public interest and encourage participation. Moreover, the similarity in perception between two genders indicates that there is no need to take tailored approaches to each gender’s perceptions and willingness level.

Every single research has its flaws no matter how much thought and consideration are given to research plans, and ours is no exception [73]. There are some limitations as to the gender distribution and online survey methods, which could be further improved. Firstly, out of 398 observations, female respondents vastly outnumbered their male counterparts, with 308 for the former and 90 for the latter. However, we applied the Mann–Whitney U test for each group to process data and made proper comparisons because the datasets for males and females are not normally distributed. Secondly, online surveys have some inherent setbacks in terms of data collection processes. A lack of direct careful supervision while respondents complete the questionnaire may lead to miscommunication, wording, and misunderstanding that potentially influence participants’ responses. To minimize and/or control this possibility, before delivering our online survey on a large scale, we formed a focus group [74] and directly interviewed them based on the questionnaire for continuous revision to produce an optimal version and resolve detected concerns immediately. In addition, we also worked collaboratively with a company specializing in online survey delivery to ensure that our survey can reach a desirable number of participants to be sufficiently large for data process and generalization.

Although this research was carried out in HCMC, the largest city in Vietnam of nearly 9 million residents, it may be slightly unrepresentative of Vietnam because of the marked difference in socio-cultural and socioeconomic factors and inherent features among cities. To broaden potential readers’ interest and policy implications, the similar study should be conducted to continuously expand our research areas with larger sample sizes associated with more diverse observations for outcomes that are representative of other regions in Vietnam.

## 6. Conclusions

Young adults’ social habits and daily activities render them prone to COVID-19 infection, compared to other segments of the population. Be that as it may, recent research indicates residents’ tendency to opt out of vaccines. This study aims to advance the understanding of adults’ perceptions and reasons behind their intentions on vaccination program participation using descriptive statistics methods and the Bayesian regression model. The results show that young adults believed that the COVID-19 pandemic only had a moderate impact on their lives in different aspects in general. Beyond our expectations, the statistics suggest that most of the young adults surveyed highly value vaccine-related dimensions in repelling the pandemic and expressed satisfaction with the Vietnamese government’s tough preventive measures and strict enforcement of regulations. Over four-fifths of our respondents were willing to get vaccinated against COVID-19. Furthermore, there was a subtle difference between male and female participants with regard to their perceptions of COVID-19 and decisions on vaccination, which confirms and/or helps policymakers to have a broader view and guarantee similarity in policy effectiveness between two genders. While getting young adults vaccinated can effectively contribute to controlling the pandemic, future research into the willingness to get vaccinated of other demographic groups will provide a more comprehensive picture of the entire population. While our empirical findings reconfirm those of many previous works, our research findings suggest that the government should focus on transparency in official information on COVID-19 vaccines and prioritize vaccines of the highest safety level to allay fears of side effects, which allows for the most appropriate policy formulation and implementation to encourage public participation in vaccination programs.

## Figures and Tables

**Table 1:** Summary of the literature on the COVID-19 vaccine acceptance rate.

Study	Country	Findings
Biasio et al., (2021) [16]	Italy	In total, 90% of respondents are willing to take the vaccine.
Cahapay (2021) [21]	Philippine	A majority of K-12 teachers are not willing to take the vaccine, because of uncertainty about the effects of the vaccine.
Dodd et al., (2021) [17]	Australia	In total, 85.5% of respondents are willing to take the vaccine.
Dong et al., (2020) [44]	China	Vaccines with high effectiveness, long protective duration, few side effects, and manufactured overseas are preferred.
García and Cerda (2021) [40]	Chile	Public acceptance toward the COVID-19 vaccine depends on the efficiency of the government at handling the pandemic.
Guidry et al., (2021) [18]	USA	Perceived benefits of the vaccines, age and race/ethnicity are among the main determinants of COVID-19 vaccination.
Harapan et al., (2021) [45]	Indonesia	Vaccine efficacy is important in determining the selection of COVID-19 vaccines.
Kadoya et al., (2021) [15]	Japan	In total, 47% respondents are willing to take the vaccine; 22% are not willing and 31% are indecisive.
McPhedran and Toombs (2021) [19]	UK	Vaccine efficacy is important in determining the selection of COVID-19 vaccines.
Neumann-Böhme et al., (2020) [46]	Europe	Vaccination willingness varies across countries, ranging from 62% in France to 80% in Denmark.
Qin, Wang and Ni (2021) [47]	China	In total, 79% of respondents are willing to get vaccinated and the average willingness to pay for a shot is 130.45 yuan. Willingness to get vaccinated decreases with age.

**Table 2:** Variables and definitions.

Code Variables	Terms Used in the Paper	Question	Variables Definitions
VacciDeci	Vaccination decision	C18: What is your decision on COVID-19 vaccination? (This is an important question, please read carefully before answering)	Binary variable. Decision of respondents in getting COVID-19 vaccination. 1 = Yes; 0 = No
Incomeff	Income effect	A1: How is the COVID-19 affecting your family’s income?	The level of COVID-19 effects on respondents’ income. Variable has 5 values. 1 = Very low; 2 = Low; 3 = Medium; 4 = High; 5 = Very high
Socialeff	Social effect	A2: How is the COVID-19 affecting your daily habit? [travelling, shopping, hanging out]?	The level of COVID-19 effects on respondents’ daily habits. Variable has 5 values. 1 = Very low; 2 = Low; 3 = Medium; 4 = High; 5 = Very high
Workeff	Work effect	A3: How is the COVID-19 affecting your work, your job [online working, jobless...]?	The level of COVID-19 effects on respondents’ job. Variable has 5 values. 1 = Very low; 2 = Low; 3 = Medium; 4 = High; 5 = Very high
Danger	Danger	A5: Your general assessment of the danger of COVID-19 pandemic on the health, economy and life of the country and the world.	Respondents’ general assessment of the Danger of COVID-19 pandemic on the health, economy and life of the country and the world. Variable has 5 values. 1 = Very safe; 2 = Safe; 3 = Neutral; 4 = Dangerous; 5 = Very dangerous
Infectprob	Infection probability	A6: In your opinion, what is the probability of being infected COVID-19 in the current situation of Vietnam?	The likelihood of getting COVID-19 in the current situation of Vietnam. Variable has 5 values. 1 = Very low; 2 = Low; 3 = Medium; 4 = High; 5 = Very high
VacciImport	Perceived importance of vaccines	A8: What is the level of vaccine importance in COVID-19 control?	The importance level of vaccine in COVID-19 control. Variable has 5 values. 1 = Not important; 2 = Less important; 3 = Normal; 4 = Important; 5 = Very important
SideeffeImport	Perceived importance of Side effects	A10: How important is vaccine side effects in your decisions on vaccination?	The importance level of effect side in respondents’ decision to get vaccinated. Variable has 4 values. 1 = Not important; 2 = Less important; 3 = Important; 4 = Very important
Gender	Gender	A14: What is your gender?	Gender of respondents. Variable has 2 values. 1 = Male; 0 = Female.

**Table 3:** Students’ perceptions of COVID-19 impacts and vaccines’ importance and dimensions.

		N	Mean	Std. Deviation	Std.Error	Min	95% Confidence Interval for Mean		Max	Range
							Lower Bound	Upper Bound		
Perceived effects	Income effect	398	3.34	0.949	0.048	1	3.25	3.44	5	4
	Habit effect	398	3.34	0.965	0.048	1	3.25	3.44	5	4
	Work effect	398	3.27	1.123	0.056	1	3.16	3.38	5	4
	General effect	398	3.42	0.896	0.045	1	3.33	3.51	5	4
Perceived risks	Infection probability	398	3.18	0.93	0.047	1	3.08	3.27	5	4
	Danger level	398	4.62	0.614	0.031	1	4.56	4.68	5	4
	Ensured safety level	398	4.57	0.642	0.032	2	4.50	4.63	5	3
Perceived importances related to vaccines	Vaccine importance	398	4.74	0.522	0.026	3	4.69	4.79	5	2
	Origin importance	398	3.23	0.755	0.038	1	3.15	3.30	4	3
	Side-effect importance	398	3.4	0.763	0.038	2	3.32	3.47	4	2
	Price importance	398	3.84	0.489	0.025	1	3.79	3.89	4	3
	Effective importance	398	3.96	0.196	0.01	3	3.94	3.98	4	1
	Convenience importance	398	3.3	0.554	0.028	1	3.25	3.36	4	3
Decision	Vaccination acceptance (yes/no)	398	0.8342	0.37240	0.01867	0	0.7975	0.8709	1	1

**Table 4:** Differences in perception and decision between two genders.

Comparison between Genders		Male						Female						Sig. (Man–Whitney U Test)
		N	Mean	Std. Deviation	Std. Error	Min	Max	N	Mean	Std. Deviation	Std. Error	Min	Max	
Perceived effects	Income effect	90	3.41	1.101	0.116	1	5	308	3.32	0.901	0.051	1	5	0.443
	Habit effect	90	3.34	1.072	0.113	1	5	308	3.34	0.933	0.053	1	5	0.817
	Work effect	90	3.39	1.129	0.119	1	5	308	3.23	1.120	0.064	1	5	0.304
	General effect	90	3.42	0.994	0.105	1	5	308	3.42	0.867	0.049	1	5	0.903
Perceived risks	Infection probability	90	2.93	1.089	0.115	1	5	308	3.25	0.868	0.049	1	5	0.021
	Danger level	90	4.58	0.719	0.076	1	5	308	4.63	0.581	0.033	2	5	0.798
	Ensured safety level	90	4.74	0.510	0.054	3	5	308	4.52	0.668	0.038	2	5	0.002
Perceived importances related to vaccines	Vaccine Importance	90	4.67	0.653	0.069	3	5	308	4.76	0.476	0.027	3	5	0.480
	Origin importance	90	3.23	0.808	0.085	1	4	308	3.23	0.740	0.042	1	4	0.748
	Side effect importance	90	3.28	0.821	0.087	2	4	308	3.43	0.743	0.042	2	4	0.121
	Price importance	90	3.73	0.650	0.069	1	4	308	3.87	0.427	0.024	1	4	0.052
	Effective importance	90	3.97	0.181	0.019	3	4	308	3.96	0.201	0.011	3	4	0.706
	Convenience importance	90	3.14	0.628	0.066	1	4	308	3.35	0.523	0.030	2	4	0.008
Decision	Vaccination acceptance (yes/no)	90	0.8556	0.353	0.03726	0.00	1.00	308	0.8279	0.37806	0.022	0.00	1.00	0.536

**Table 5:** Summary of the estimated coefficients from hierarchical Vaccination Decision model.

				Percentile Statistics						
Variables	Mean	se_Mean	Sd	2.5%	25%	50%	75%	97.5%	*n_eff*	*Rhat*
a_VacciDeci	−0.41	0.02	1.61	−3.57	−1.50	−0.43	0.67	2.81	7577	1
b_Incomeff_VacciDeci	−0.07	0.00	0.19	−0.44	−0.20	−0.07	0.06	0.30	10,367	1
b_Socialeff_VacciDeci	−0.14	0.00	0.17	−0.48	−0.25	−0.14	−0.02	0.19	11,718	1
b_Workeff_VacciDeci	0.09	0.00	0.15	−0.20	−0.01	0.09	0.19	0.38	9857	1
b_Danger_VacciDeci	−0.35	0.00	0.27	−0.90	−0.53	−0.35	−0.17	0.15	8785	1
b_Infectprob_VacciDeci	0.07	0.00	0.16	−0.23	−0.03	0.07	0.18	0.39	11,633	1
b_VacciImport_VacciDeci	1.26	0.00	0.25	0.77	1.09	1.25	1.43	1.76	10,099	1
b_SideeffeImport_VacciDeci	−0.58	0.00	0.22	−1.02	−0.73	−0.57	−0.42	−0.16	10,730	1
b_Gender_VacciDeci	0.39	0.00	0.38	−0.33	0.12	0.38	0.64	1.16	9546	1

## Data Availability

The data presented in this study are openly available in the Mendeley platform at https://data.mendeley.com/datasets/3rc6fbdbbp/1 (accessed on 10 May 2021). The data are available under the CC BY 4.0 license.
